# 

**Published:** 1854-01

**Authors:** 


### HALL'S

### JOURNAL OF HEALTH,

### FOR 1854.

HEALTH IS A DUTY.—ANON.

"MEN CONSUME TOO MUCH FOOD AND TOO LITTLE PURE AIR; THEY TAKE TOO MUCH MEDICINE AND TOO LITTLE EXERCISE."—*Ed.*

"I labor for the good time coming, when sickness and disease, except congenital, or from accident, will be regarded as the result of ignorance or animalism, and will degrade the individual, in the estimation of the good, as much as drunkenness now does.—IBID.

EDITED BY

W. W. HALL, M.D.,

42 IRVING PLACE, N. Y.

Surgeon Gen'l's Office LIBRARY. Washington, D.C. 18491

32 Cat

VOL. I.

New York:

PUBLISHED BY HENRY B. PRICE,

No. 8 EVERETT HOUSE, UNION SQUARE.

1854.

